# Triplet Energy Transfer Mechanism of Ternary Organic Hybrid Thin Films of PFO/MEH-PPV/CsPbBr_3_ Perovskite Quantum Dots

**DOI:** 10.3390/nano10112094

**Published:** 2020-10-22

**Authors:** Bandar Ali Al-Asbahi, Saif M. H. Qaid, Hamid M. Ghaithan, Abdullah S. Aldwayyan

**Affiliations:** 1 Department of Physics & Astronomy, College of Science King Saud University, Riyadh 11451, Saudi Arabia; sqaid@ksu.edu.sa (S.M.H.Q.); 436107632@student.ksu.edu.sa (H.M.G.); dwayyan@ksu.edu.sa (A.S.A.); 2 Department of Physics, Faculty of Science, Sana’a University, Sana’a P.O. Box 12544, Yemen; 3 Department of Physics, Faculty of Science, Ibb University, Ibb P.O. Box 70270, Yemen; 4 King Abdullah Institute for Nanotechnology, King Saud University, Riyadh 11451, Saudi Arabia; 5 K.A.CARE Energy Research and Innovation Center at Riyadh, P.O. Box 2022, Saudi Arabia

**Keywords:** triple energy transfer mechanism, Förster resonance energy transfer, donor/acceptor, perovskite quantum dots, ternary hybrid thin films

## Abstract

The triplet energy transfer mechanism of novel poly(9,9-di-*n*-octylflourenyl-2,7-diyl) (PFO)/poly[2-methoxy-5-(2-ethylhexyloxy)-1,4-phenylenevinylene] (MEH-PPV)/CsPbBr_3_ perovskite quantum dot (PQD) hybrid thin films was comprehensively investigated. The concentrations of PFO and MEH-PPV in all the specimens were fixed, while the PQD content was varied with various weight ratios and premixed by a solution blending method before it was spin-coated onto glass substrates. The triplet non-radiative Förster resonance energy transfers (FRETs) in the PFO/MEH-PPV/PQDs ternary blend, the dual FRET from PFO to both PQDs and MEH-PPV, and the secondary FRET from PQDs to MEH-PPV were observed. The values of the Förster radius (*R*_o_) of FRET from PFO to MEH-PPV in the presence of various PQD contents (Case I) increased from 92.3 to 104.7 Å, and they decreased gradually from 68.0 to 39.5 Å for FRET from PFO to PQDs in the presence of MEH-PPV (Case II). These *R*_o_ values in both cases confirmed the dominance of FRET in ternary hybrid thin films. Upon increasing the PQD content, the distance between the donor and acceptor molecules (*R_DA_*) and the conjugation length (A_π_) in both cases gradually decreased. The small values of *R*_o_, *R_DA_*, and A_π_ with a decrease in the energy transfer lifetime (*τ_ET_*) due to an increase in the PQD contents in both Cases I and II confirmed the efficient FRET in the hybrid. To prevent intermolecular transfer in PFO, the concentrations of MEH-PPV (Case I) and PQDs (Case II) should be decreased to a range of 0.57–0.39 mM and increased in the range of 1.42–7.25 mM.

## 1. Introduction

Optoelectronic devices, such as photovoltaic sensors, solar cells, and organic light-emitting diodes (OLEDs), that are based on a donor/acceptor binary hybrid of conjugated polymers have attracted the attention of many researchers because they are inexpensive, lightweight, and easy to prepare. Additionally, they exhibit high mechanical flexibility and are an ecofriendly energy source [1,2,3,4]. 

To improve the performance of optoelectronic devices, such as OLEDs, several techniques have been employed [5,6]. One of the recent techniques is the utilization of the Förster resonance energy transfer (FRET) effect [7,8,9,10]. The utilization of FRET between appropriate donor/acceptor pairs is still an evolving field of photon harvesting, and it could mitigate the deficiencies related to traditional light-conversion techniques through the manufacture of OLEDs. Furthermore, the characteristics of light emitted from OLEDs can be tuned by changing the donor/acceptor ratio in FRET-based hybrids [11]. Nevertheless, the main requirements for efficient FRET include the following: a good spectral overlap between the acceptor absorption and donor emission, the proximity of the energy levels of the acceptor to that of the donor, and the utilization of a donor with high photoluminescence quantum yield in the system. Hence, an appropriate donor/acceptor combination is crucial to achieving FRET-based OLEDs with high performance. In our recent reports, appropriate donor/acceptor combinations, such as PFO/MEH-PPV [12], Cs_4_PbBr_6_/MEH-PPV [13], and PFO/CsPbBr_3_ perovskite quantum dots (PQDs) [14], were employed to achieve efficient FRET.

Recently, it was found that the incorporation of metal oxide nanostructured materials into the donor/acceptor blends could improve the FRET properties, which will consequently improve the performance of OLEDs [7,8,12]. However, the incorporation of metal oxide nanostructured materials into an organic donor/acceptor blend is limited by their insolubility in organic solvents, which will result in high agglomeration in the blends. PQDs, such as CsPbX_3_ (X= Cl, I, and Br), are among the new important alternatives to metal oxide nanomaterials in optoelectronic applications. Their promising potentials are attributable to their features, such as solubility in common organic solvents; broad emission wavelength through the visible spectrum; short radiative lifetime; high photoluminescence quantum yield; very narrow emission bandwidth; many options for shape modification; and significant optical properties, including stimulated emission, nonlinear absorption, and blinking behavior [15,16,17].

A novel approach toward tuning the FRET properties of a common PFO/MEH-PPV binary blend was demonstrated in this study. This approach involved the incorporation of a third material, which functioned simultaneously as the acceptor and donor. This material is CsPbBr_3_ PQDs, in which the lowest unoccupied molecular orbital (LUMO) and highest occupied molecular orbital (HOMO) levels were between those of the donor (PFO), while the energy levels of the acceptor (MEH-PPV) were between those of PQDs. Therefore, PQDs functioned as an acceptor with PFO and as a donor with MEH-PPV. Thus, a triplet FRET was achievable in the current novel ternary blend, a dual FRET from PFO to both PQDs and MEH-PPV, and a secondary FRET from PQDs to MEH-PPV, as would be demonstrated. Moreover, to the best of our knowledge, this will be the first report on triplet FRET in a PFO/PQDs/MEH-PPV ternary blend. The photophysical properties and energy transfer mechanism between PFO and MEH-PPV were also investigated here by incorporating various weight ratios of PQDs.

## 2. Materials and Methods

### 2.1. Materials 

Each PFO (Mw = 58,200 g/mol), MEH-PPV (Mw = 40,000 g/mol), and toluene were purchased from Sigma Aldrich (Saint Louis, MO, USA). High-quality green-emitting CsPbBr_3_ PQDs in solution form (dissolved in toluene with a concentration of 10 mg/mL (were obtained from Quantum Solutions LLC, King Abdullah University of Science and Technology (KAUST), Thuwal, Saudi Arabia. All materials were utilized without further purification.

### 2.2. Methods and Characterizations

First, the glass substrates with 1 cm × 2 cm dimensions were cleaned, as reported in our recent work [14]. Next, PFO and MEH-PPV were dissolved separately in toluene with concentrations of 30 and 0.5 mg/mL, respectively, as stock solutions. Thereafter, the three materials (PFO, MEH-PPV, and PQDs) were premixed by a solution blending method with various weight ratios of PQDs (0.0, 0.5, 1.0, 3.0, 5.0, 7.0, and 10 wt.%). For this purpose, the concentrations of PFO and MEH-PPV in all the samples were fixed at 15 and 0.5 mg/mL, respectively, while that of PQDs varied at 3.80, 2.67, 1.68, 0.80, 0.15, and 0.08 mg/mL. Afterward, 50 mL of each sample was dropped onto the cleaned glass substrates and followed by spin-coating at 3000 rpm for 30 s to afford homogenous thin films. 

Ultraviolet–visible (UV–vis) spectrometry utilizing a JASCO V-670 spectrometer (JASCO, Cremella, Italia) and a spectrofluorometry (JASCO FP-8200) (JASCO, Cremella, Italia) were employed to analyze the absorption and photoluminescence spectra, respectively. Afterward, the data of the absorption and emission were analyzed by OriginLab software, version 8.0 (Northampton, MA, USA) to determine the parameters of energy transfer. All the measurements were performed in an ambient atmosphere.

## 3. Results

### 3.1. Optical Properties

**Figure 1:**
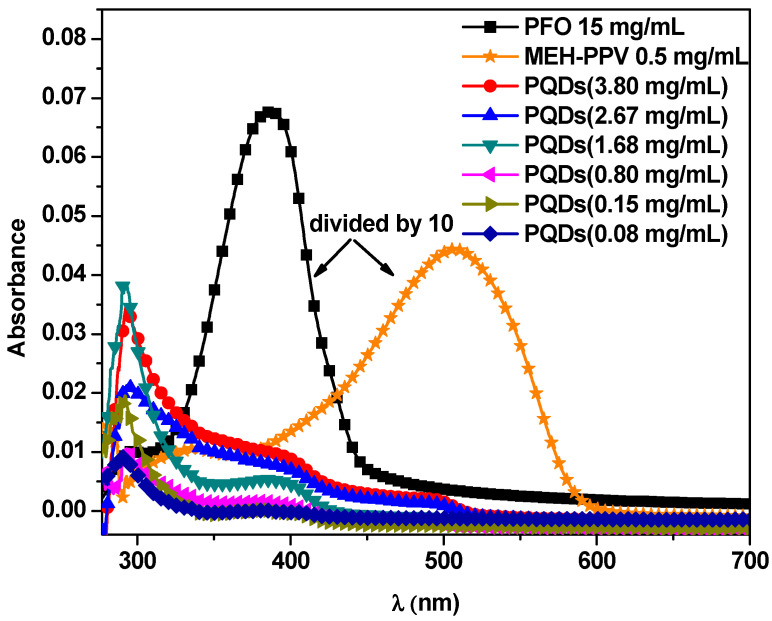
Absorption spectra of pristine PFO, MEH-PPV, and perovskite quantum dots (PQDs) at specific concentrations that were equal to the ratios utilized for the hybrids.

Figure 1 shows the optical absorption spectra of pristine PFO, MEH-PPV, and PQDs (at various equivalent concentrations to those ratios utilized in the hybrids). The PFO and MEH-PPV spectra exhibited maximum peaks at 385 and 508 nm with shoulders at 294 and 340 nm, respectively. The spectra of PQDs exhibited three peaks at 291, 392, and 495 nm, at which the absorbance decreased as its concentration decreased. Compared to the maximum peaks of PFO and PQDs, the π–π^*^ absorption band of MEH-PPV was broader within the visible region with bathochromic shifts of 123 and 217 nm compared to PFO and PQDs, respectively. These shifts could be ascribed to a further extension of the π-conjugation through the molecular backbone [18,19].

**Figure 2:**
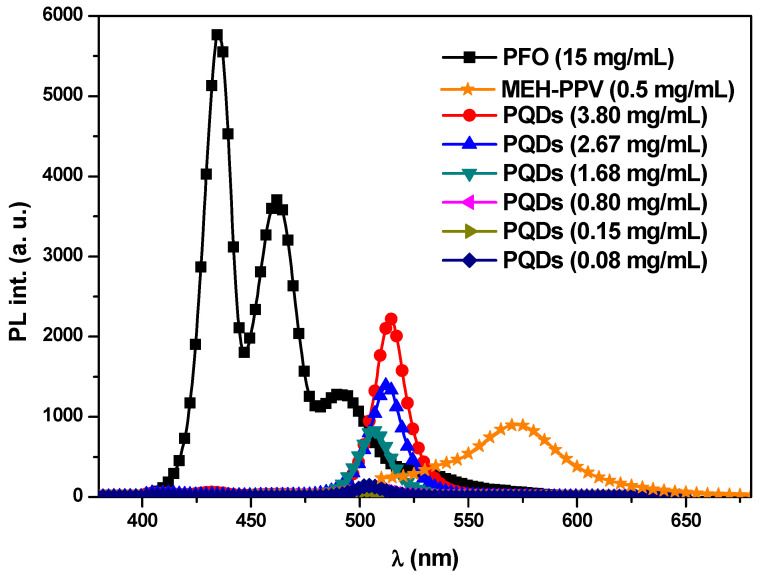
Photoluminescence spectra of pristine PFO, MEH-PPV, and PQDs at specific concentrations that were equal to the ratios utilized for the hybrids.

The fluorescence spectra of pristine PFO, MEH-PPV, and PQDs (at various equivalent concentrations to the ratios utilized in the hybrids) with an excitation wavelength at 355 nm are shown in Figure 2. Regarding the pristine PFO, three main peaks were detected at 492, 462, and 435 nm, which corresponded to the 0→2, 0→1, and 0→0 vibronic transitions, respectively, with a shoulder peak at ~530 nm that corresponded to the formation of keto defects throughout the photooxidation process [20,21]. The maximum peaks at 572 and 514 nm were attributed to the 0→0 vibronic transitions of MEH-PPV and PQDs, respectively. The dramatic decrease in the emission intensity of PQDs as its concentration decreased could be attributed to the aggregation. Moreover, the higher intensity of PFO than those of MEH-PPV and PQDs indicated that the excitation wavelength of 355 nm was dominantly absorbed by PFO.

**Figure 3:**
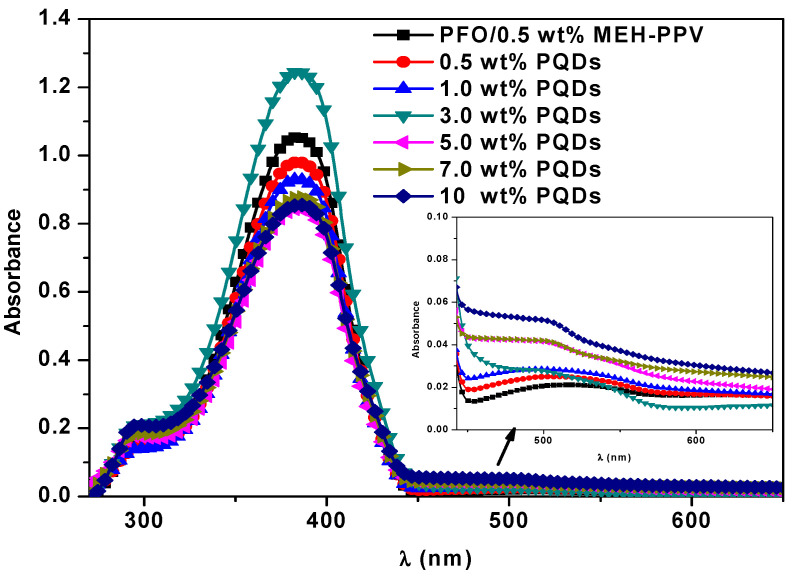
Absorption spectra of the hybrid thin films of PFO/MEH-PPV with various PQD contents.

Figure 3 shows the absorption spectra of the hybrid thin films of PFO/MEH-PPV with various PQD contents. For the PFO/MEH-PPV blend with various ratios of PQDs, the maximum peak at 385 nm dramatically decreased without any shift, and the absorbance peak of the shoulder at 294 nm increased slightly. Moreover, the absorbance in the range of 450–600 nm (Figure 3 inset) slightly increased with an increase in the PQD content. Furthermore, a comparison of the ternary blend and the individual components (Figure 1) confirmed the absence of any new absorbance peak when the PQDs content was increased in the PFO/MEH-PPV binary blend, implying that no dimer had been formed in the ternary hybrids. 

**Figure 4:**
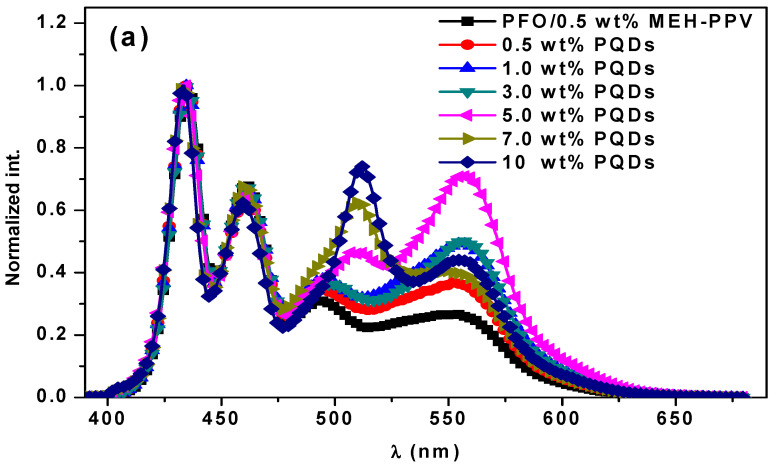

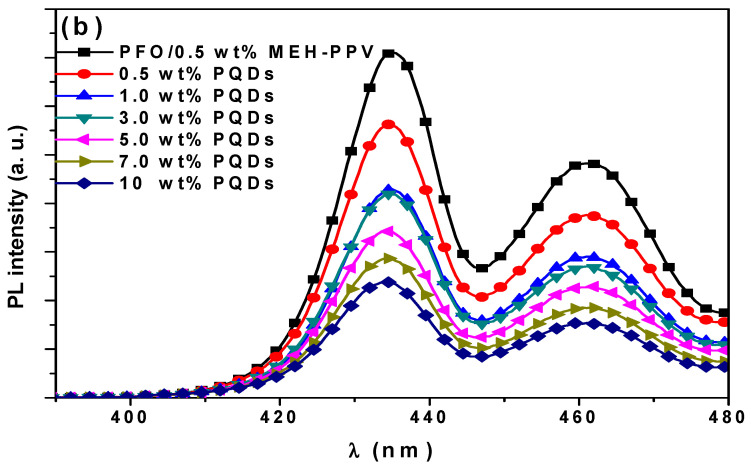
(**a**) Normalized emission intensity of the hybrid thin films of PFO/MEH-PPV with various PQD contents, (**b**) emission intensity of the hybrid thin films of PFO/MEH-PPV with various PQD contents in the range 390–480 nm.

**Figure 5:**
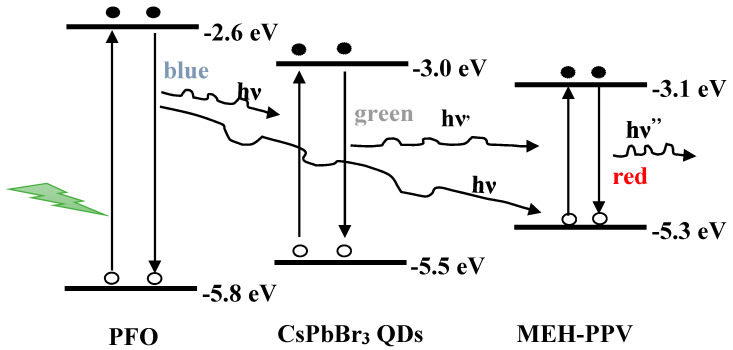
Scheme of the triplet Förster resonance energy transfer (FRET) in the PFO/MEH-PPV/PQDs hybrids.

Figure 4a shows the emission spectra of the PFO/MEH-PPV hybrid thin film without and with varying ratios of PQDs. The nonradiative energy transfer (the Förster type) from PFO to MEH-PPV was observed in our recent study [12]. With an increment in the PQDs, the intensities relating to both PQDs (at ~500 nm) and MEH-PPV (at ~555 nm) increased with a gradual redshift in the peak of PQDs. The redshift in the peak of PQDs was attributed to the existence of radiative migration due to self-absorption, as proved by other systems in previous reports [12,22]. The emission peaks related to the PFO (at 435 and 462 nm) were magnified in Figure 4b to clearly display the effect of the PQD content on their intensities. It could be observed that the incorporation of PQDs into the PFO/MEH-PPV hybrid systematically reduced the emission intensity peaks of PFO. This behavior was attributed to the dual non-radiative FRET from PFO to both PQDs and MEH-PPV in the ternary blend. However, the significant increase in the intensity of MEH-PPV as PQDs increased (Figure 4a) implied a secondary FRET from the molecules of PQDs to those of MEH-PPV. Furthermore, as shown in Figure 4a, the opposite trend was observed in the emission intensity of MEH-PPV as more PQDs (>5 wt.%) were added to the hybrid thin films. This is because it was easier to continue transferring the whole nonradiative energy from the excited PFO molecules to a lot of the PQD molecules than to MEH-PPV (Figure 5). Thereafter, the PQD molecules contributed their energy to the MEH-PPV molecules through a secondary FRET mechanism. This finding was also supported by the offset energy levels of the ternary materials (Figure 5), where the MEH-PPV energy levels were between those of PQDs. In the PFO/MEH-PPV/PQDs ternary blend, a light energy irradiates the PFO and then its oscillating dipole can be produced and resonated with the oscillating dipole of both MEH-PPV and PQDs. Consequently, the excited state energy can be transferred through space (dipole–dipole interaction) from the PFO to both MEH-PPV and PQDs. Once the PFO comes back to the ground state, a non-radiative energy transfer takes place, and both MEH-PPV and PQDs are brought to the excited state. Meanwhile, by the same way, a secondary energy transfer through dipole–dipole interaction can be occurred from the PQDs to MEH-PPV. 

### 3.2. FRET Parameters of the Ternary Hybrid Thin Films

**Figure 6:**
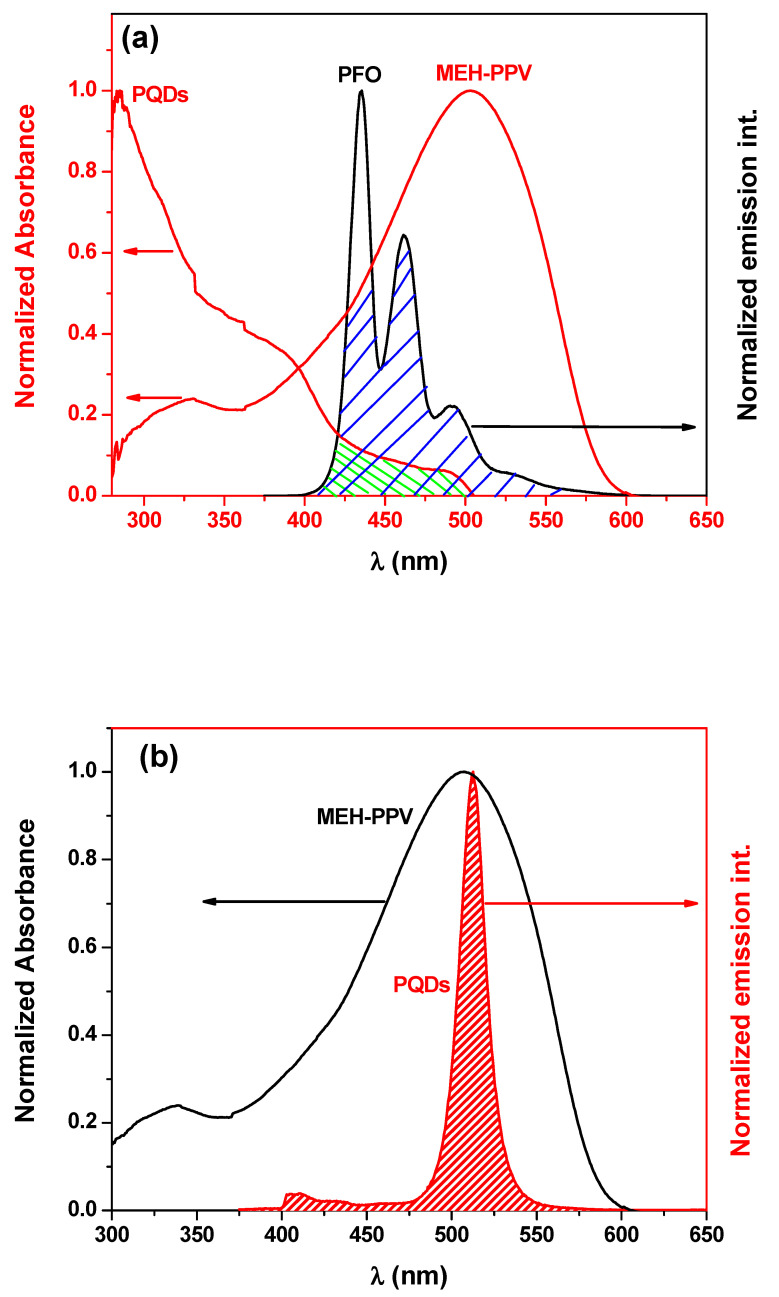
(**a**) Overlap between the emission spectrum of PFO with the absorption spectra of both the PQDs and MEH-PPV thin films, (**b**) overlap between the emission spectrum of PQDs and the absorption spectrum of the PFO thin films.

Triplet FRET could be initially observed from the arrangement of the energy levels of the three materials (Figure 5) as well as from the significant overlapping of their emission and absorption spectra. The dual FRET from PFO to both PQDs and MEH-PPV could be proven by the existence of HOMO and LUMO of PQDs and MEH-PPV in those of PFO (Figure 5) and from the overlapping of the emission spectrum of PFO with the absorption spectra of each of PQDs and MEH-PPV (Figure 6a). The secondary FRET from PQDs to MEH-PPV was expected from the existence of HOMO and LUMO of MEH-PPV between those of PQDs (Figure 5) and from the large overlapping of the emission spectrum of PQDs with the absorption spectrum of MEH-PPV (Figure 6b). The effect of various PQD contents on FRET from PFO to MEH-PPV (Case I) would be described in the sections below. Moreover, FRET from PFO to PQDs with a fixed amount of MEH-PPV (Case II) would also be comprehensively investigated.

#### 3.2.1. Quantum Yield (φ_DA_) and Lifetime (τ_DA_) of the Donor in the Ternary Hybrid Thin Films

Both the φ*_DA_* and τ*_DA_* values of the donor (PFO) in the ternary hybrid thin films were estimated from the intensities of the photoluminescence spectra of the PFO/MEH-PPV hybrid without and with various PQD content ratios. As reported elsewhere [8,23,24], the following formula can be used to determine both φ*_DA_* and *τ_DA_* based on the donor emission intensities *I_D_* and *I_DA_* in the absence and presence of acceptors, respectively:

(1) $$\frac{I_{D}}{I_{DA}}=\frac{\varphi_{D}}{\varphi_{DA}}=\frac{\tau_{D}}{\tau_{DA}}$$

The results are listed in Table 1. These values were systematically reduced as the PQD content increased, indicating that radiative energy transfer was possible in the ternary system. Moreover, since these values (φ*_DA_* and *τ_DA_*) were smaller than those of the pristine donor (φ*_D_* = 0.72 and τ*_D_* = 364 ps [23]), an efficient non-radiative energy transfer could occur in the binary hybrid thin films of PFO/MEH-PPV in the presence of PQDs. 

#### 3.2.2. Stern–Volmer (k_SV_) and the Quenching Rate (k_q_) Constants

The concentration of the acceptor (MEH-PPV) that was required to reduce the PFO emission intensity of the hybrid thin films to half its value (A_1/2_) could be estimated by determining the *k_SV_* values of the energy transfer from PFO to MEH-PPV (Case I). The systematic increase in the *k_SV_* values upon increasing the PQD contents indicated that half of the emission was quenched as A_1/2_ gradually decreased from ~15.0 to 0.80 µM, as shown in Table 1. Conversely, the adequate incorporation of PQDs into the hybrid (PFO/MEH-PPV) and the good quality of the interface between the donor and acceptor with increasing PQDs were confirmed by the gradual increase in the *k_q_* values (Table 1). Moreover, these *k_q_* values were much greater than the minimum value of efficient quenching (1 × 10^10^ M^−1^S^−1^) [24]. 

**Figure 7:**
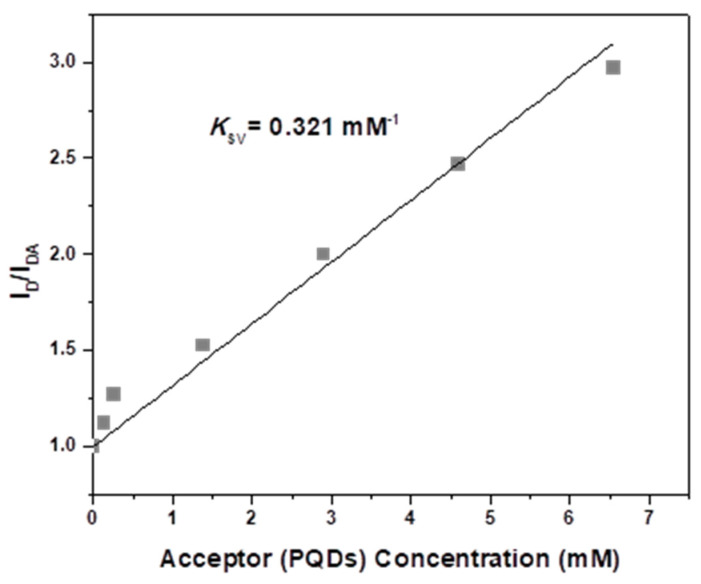
Stern–Volmer plot for the emission quenching of PFO with various PQD contents.

Furthermore, the number of PQDs could be determined to reduce 50% of the emission intensity of the PFO thin film in the presence of MEH-PPV. For this purpose, the *k_SV_* value of FRET from PFO to PQDs (0.321 mM^−1^) (Case II) was obtained from the Stern–Volmer plot (Figure 7). This finding suggested that the concentration of PQDs should be 3.11 mM to reduce the emission intensity of the donor to half its value. In this case, the value of *k_q_* was 9.28 × 10^11^ M^−1^s^−1^, indicating the adequate mixing of PQDs with the conjugated polymers. 

#### 3.2.3. Energy Transfer Distance and Lifetime

As applied in previous reports [24,25], the critical distance (Förster radius, *R*_0_) was calculated by the following Equation:

(2) $$R_{o}^{6}=\frac{8.79\times 10^{-5}\beta^{2}\varphi_{D}}{n^{4}}\int^{}F_{D}\left(\lambda \right)\varepsilon_{A}\left(\lambda \right)\;\lambda^{4}d\lambda =\frac{8.79\times 10^{-5}\beta^{2}\varphi_{D}}{n^{4}}\;J\left(\lambda \right)$$

where *n*, *β*^2^, $\varphi_{D},\;\lambda ,\;\varepsilon_{A}\left(\lambda \right)$, and *F_D_* (λ) are the solvent refractive index, orientation factor (0.67 for isotropic media), donor quantum yield, wavelength, molar decadic extinction coefficient of the acceptor, and normalized spectral distribution of the donor, respectively. The values of *J*(λ) and *R*_o_ are presented in Table 1 for Case I (FRET from PFO to MEH-PPV in the presence of PQDs) and Table 2 for Case II (FRET from PFO to PQDs in the presence of MEH-PPV). In Case I, *J*(λ) increased from 7.32 × 10^16^ to 15.6 × 10^16^ M^−1^cm^−1^nm^4^. Thus, *R*_o_ increased from 92.3 to 104.7 Å. In Case II, *J*(λ) gradually decreased from 1.17 × 10^16^ to 0.045 × 10^16^ M^−1^cm^−1^nm^4^. Thus, *R*_o_ reduced from 68.0 to 39.5 Å. *R*_o_ in both cases confirmed the dominant FRET in the hybrid thin films [26,27]. 

Based on the *R*_o_ values and emission intensities of the donor with and without the acceptor, the distance between the donor and acceptor molecules (*R_DA_*) in both cases could be calculated [8]. As presented in Table 2 and Table 3, the values of *R_DA_* gradually decreased from 130.6 to 90.9 Å (Case I) and 78.8 to 34.3 Å (Case II) as the PQD content increased. The decrease in *R_DA_* with increasing PQDs indicated that PQDs decreased the distance between the donor and acceptor molecules. Moreover, the smaller values of *R*_o_ and *R_DA_* in Case II than in Case I was attributed to the favorable increase in the surface area in addition to the convergence of their energy levels, as presented in Figure 5, thus easing FRET. 

Another proof of efficient FRET in the current system is the decrease in the energy transfer lifetime (*τ_ET_*) with increasing the PQD contents in both Cases I and II. The following formula was used to determine the *τ_ET_* values [24]:

(3) $$\tau_{ET}=\frac{1}{\left[A\right]k_{q}}$$

where [*A*] is the acceptor concentration. The *τ_ET_* values reduced significantly from 2775 to 148 ps (Case I) and 7810 to 164 ps (Case II), as demonstrated in Table 2 and Table 3, respectively, indicating the efficient FRET in the ternary PFO/MEH-PPV/PQDs blend.

#### 3.2.4. Critical Concentration of the Acceptor (A_o_) and Conjugation Length (A_π_)

To prevent intermolecular transfer in the donor (PFO), the concentration of the acceptor (PQDs and MEH-PPV) should be limited, specifically less than A_o_ [13,28], where A_o_ is the concentration of the acceptor at which 76% of the energy could be transferred. In Case I, A_o_ of MEH-PPV decreased in the range of 0.57–0.39 mM with the addition of PQDs, as shown in Table 1. As *R*_0_ decreased (Case II), A_o_ of PQDs increased in the range of 1.42–7.25 mM, as listed in Table 3. Moreover, A_π_ could be calculated employing both the non-radiative rate constant (*k_nr_*) and the radiative rate constant (*k_r_*). When PQD was added, there was no change in k_r_ (~2.08 ns^−1^), although there was a significant increase in k_nr_ from 1.17 to 7.56 ns^−1^, as listed in Table 1. Subsequently, A_π_ reduced with a further addition of PQDs (Table 1). This decrease in A_π_ indicated that PQDs had reduced the distance between the molecules of MEH-PPV and PFO, thus enhancing the surface area of the hybrid thin films, as previously suspected.

**Figure 8:**
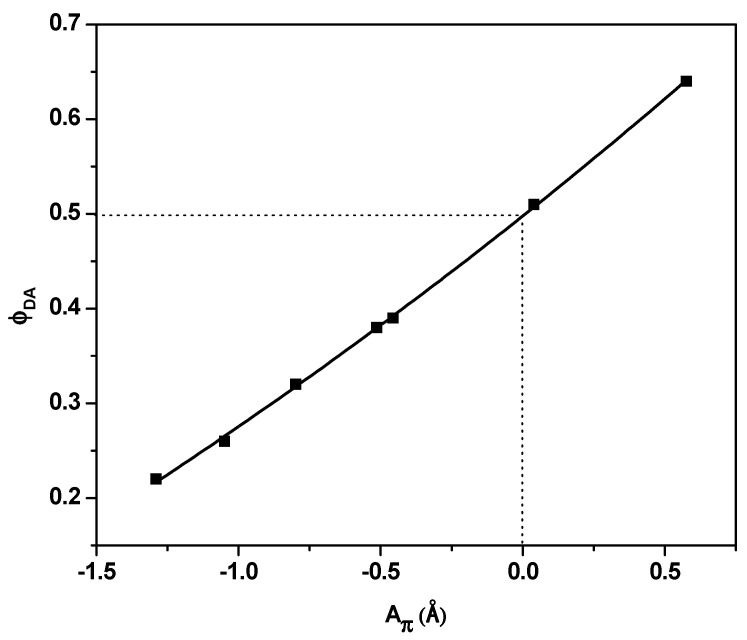
Photoluminescence quantum yield versus A_π_ with varying PQD contents.

The exponential relationship between A_π_ and φ*_DA_* (Figure 8) indicated that the addition of PQDs could produce hybrids that are highly fluorescent. Theoretically, when *k_r_* = *k_nr_*, A_π_ = 0 and φ*_DA_* = ~0.5. This finding is consistent with the experimental results shown in Figure 8.

## 4. Discussion and Conclusion

The addition of PQDs as the third component of a PFO/MEH-PPV binary blend was crucial to facilitating triplet FRET as well as tuning the FRET parameters. Dual FRET from PFO to both MEH-PPV and PQDs and secondary FRET from PQDs to MEH-PPV were confirmed. The efficiency of FRET was demonstrated by decreases in the φ*_DA_*, *τ_DA_*, and *τ_ET_* values. Moreover, the proper mixing of the three components was theoretically confirmed by the high values of *k_q_*. Further, 50% of PFO emission was quenched as MEH-PPV concentration (A_1/2_) decreased systematically from ~15.0 to 0.80 µM with increasing PQD content from 0 to 10 wt.%. The same degree of quenching could be achieved by incorporating PQDs with a concentration of 3.11 mM into the PFO/MEH-PPV binary blend. *R*_o_ in Cases I and II were in the range of 68.0–104.7 Å, confirming the significant FRET in the ternary blended thin films. The increase in the PQD content of the PFO/MEH-PPV binary blend resulted in a decrease in A_π_, thus reducing the distance between the PFO and MEH-PPV molecules. We concluded that the concentrations of PQDs and MEH-PPV in Cases I and II should be in the ranges of 1.42–7.25 mM and 0.57–0.39 mM to prevent intermolecular transfer in PFO, respectively. We believe that the utilization of the PFO/MEH-PPV/PQDs ternary blend as an active layer would aid the development of novel and promising optoelectronic devices.

## Figures and Tables

**Table 1:** Optical properties of the PFO/MEH-PPV hybrid thin film with various PQD contents.

PQDs (wt.%)	φ*_DA_*	*τ_DA_* (ps)	*k_nr_* (ns)^−1^	*k_sv_* (µM)^−1^	*k_q_* × 10^15^ (M.S)^−1^	A_π_ (Å)	A_0_ (mM)	A_1/2_ (µM)
0	0.64	308	1.17	0.067	0.192	0.576	0.57	15.0
0.5	0.51	245	2.00	0.220	0.635	0.039	0.52	4.55
1.0	0.39	186	3.29	0.459	1.326	^−^0.456	0.48	2.18
3.0	0.38	180	3.48	0.492	1.423	^−^0.514	0.45	2.03
5.0	0.32	149	4.62	0.704	2.034	^−^0.798	0.40	1.42
7.0	0.26	125	5.94	0.948	2.739	^−^1.049	0.42	1.06
10	0.22	104	7.56	1.246	3.601	^−^1.290	0.39	0.80

**Table 2:** Parameters of energy transfer from PFO to the MEH-PPV hybrid thin films with various PQD contents.

PQDs (wt.%)	*J(λ)* × 10^16^ (M^−1^cm^−1^nm^4^)	*R*_0_ (Å)	*R_DA_* (Å)	*τ_ET_* (ps)
0	7.32	92.3	130.6	2775
0.5	8.64	94.9	110.0	839
1.0	9.96	97.2	99.7	402
3.0	11.4	99.4	100.7	375
5.0	14.6	103.6	98.9	262
7.0	13.4	102.1	92.8	195
10	15.6	104.7	90.9	148

**Table 3:** Parameters of energy transfer from PFO to the PQD hybrid thin films in the presence of a fixed amount of MEH-PPV.

PQDs (wt.%)	*J(λ)* × 10^16^ (M^−1^cm^−1^nm^4^)	*R*_0_ (Å)	*R_DA_* (Å)	*τ_ET_* (ps)	A_0_ (mM)
0.5	1.17	68.0	78.8	7810	1.42
1.0	0.68	62.2	63.8	4170	1.86
3.0	0.16	48.6	49.2	781	3.89
5.0	0.094	44.7	42.7	372	5.00
7.0	0.055	40.8	37.1	234	6.58
10	0.045	39.5	34.3	164	7.25

## References

[1] Xu X., Li Z., Wang J., Lin B., Ma W., Xia Y., Andersson M.R., Janssen R.A.J., Wang E. (2018). High-performance all-polymer solar cells based on fluorinated naphthalene diimide acceptor polymers with fine-tuned crystallinity and enhanced dielectric constants. Nano Energy.

[2] Liu C.-M., Su Y.-W., Jiang J.-M., Chen H.-C., Lin S.-W., Su C.-J., Jeng U.-S., Wei K.-H. (2014). Complementary solvent additives tune the orientation of polymer lamellae, reduce the sizes of aggregated fullerene domains, and enhance the performance of bulk heterojunction solar cells. J. Mater. Chem. A.

[3] Lu L., Zheng T., Wu Q., Schneider A.M., Zhao D., Yu L. (2015). Recent Advances in Bulk Heterojunction Polymer Solar Cells. Chem. Rev..

[4] Tseng H.-R., Phan H., Luo C., Wang M., Perez L.A., Patel S.N., Ying L., Kramer E.J., Nguyen T.Q., Bazan G.C. (2014). High-Mobility Field-Effect Transistors Fabricated with Macroscopic Aligned Semiconducting Polymers. Adv. Mater..

[5] Godlewski J. (2005). Currents and photocurrents in organic materials determined by the interface phenomena. Adv. Colloid Interface Sci..

[6] Lu J., Tao Y., D’Iorio M., Li Y., Ding J., Day M. (2004). Pure Deep Blue Light-Emitting Diodes from Alternating Fluorene/Carbazole Copolymers by Using Suitable Hole-Blocking Materials. Macromolecules.

[7] Al-Asbahi B.A., Jumali M.H.H., AlSalhi M.S. (2016). Enhanced Optoelectronic Properties of PFO/Fluorol 7GA Hybrid Light Emitting Diodes via Additions of TiO_2_ Nanoparticles. Polymers.

[8] Al-Asbahi B.A. (2017). Energy transfer mechanism and optoelectronic properties of (PFO/TiO_2_)/Fluorol 7GA nanocomposite thin films. Opt. Mater..

[9] Zhang D., Cai M., Zhang Y., Bin Z., Zhang D., Duan L. (2016). Simultaneous Enhancement of Efficiency and Stability of Phosphorescent OLEDs Based on Efficient Forster Energy Transfer from Interface Exciplex. ACS Appl. Mater. Interfaces.

[10] Pramanik A., Biswas S., Tiwary C.S., Kumbhakar P., Sarkar R., Kumbhakar P. (2020). Forster resonance energy transfer assisted white light generation and luminescence tuning in a colloidal graphene quantum dot-dye system. J. Colloid Interface Sci..

[11] Trindade F.D.J., Triboni E.R., Castanheira B., Brochsztain S. (2015). Color-Tunable Fluorescence and White Light Emission from Mesoporous Organosilicas Based on Energy Transfer from 1,8-Naphthalimide Hosts to Perylenediimide Guests. J. Phys. Chem. C.

[12] Al-Asbahi B.A., Qaid S.M., Jumali M.H.H., AlSalhi M.S., Aldwayyan A.S. (2019). Long-range dipole–dipole energy transfer enhancement via addition of SiO2/TiO2 nanocomposite in PFO/MEH-PPV hybrid thin films. J. Appl. Polym. Sci..

[13] Al-Asbahi B.A., Qaid S.M.H., Al Dwayyan A.S.A. (2020). Effect of Donor-Acceptor Concentration Ratios on Non-Radiative Energy Transfer in Zero-Dimensional Cs4PbBr6 Perovskite/MEH-PPV Nanocomposite Thin Films. Polymers.

[14] Qaid S.M.H., Al-Asbahi B.A., Ghaithan H.M., AlSalhi M.S., Al Dwayyan A.S. (2020). Optical and structural properties of CsPbBr3 perovskite quantum dots/PFO polymer composite thin films. J. Colloid Interface Sci..

[15] Meyns M., Perálvarez M., Heuer-Jungemann A., Hertog W., Ibáñez M., Nafria R., Genç A., Arbiol J., Kovalenko M.V., Carreras J. (2016). Polymer-Enhanced Stability of Inorganic Perovskite Nanocrystals and Their Application in Color Conversion LEDs. ACS Appl. Mater. Interfaces.

[16] Yakunin S., Protesescu L., Krieg F., Bodnarchuk M.I., Nedelcu G., Humer M., De Luca G., Fiebig M., Heiss W., Kovalenko M.V. (2015). Low-threshold amplified spontaneous emission and lasing from colloidal nanocrystals of caesium lead halide perovskites. Nat. Commun..

[17] Rainò G., Nedelcu G., Protesescu L., Bodnarchuk M.I., Kovalenko M.V., Mahrt R.F., Stöferle T. (2016). Single Cesium Lead Halide Perovskite Nanocrystals at Low Temperature: Fast Single-Photon Emission, Reduced Blinking, and Exciton Fine Structure. ACS Nano.

[18] Bouzayen N., Zaidi B., Mabrouk A., Chemek M., Alimi K. (2012). Density functional theory studies of new bipolar carbazole–benzothiazole: Electronic and vibrational properties. Comput. Theor. Chem..

[19] Jungsuttiwong S., Tarsang R., Sudyoadsuk T., Promarak V., Khongpracha P., Namuangruk S. (2013). Theoretical study on novel double donor-based dyes used in high efficient dye-sensitized solar cells: The application of TDDFT study to the electron injection process. Org. Electron..

[20] Al-Asbahi B.A., Jumali M.H.H., Yap C.C., Salleh M.M. (2013). Influence of TiO_2_ Nanoparticles on Enhancement of Optoelectronic Properties of PFO-Based Light Emitting Diode. J. Nanomater..

[21] List E.J.W., Guentner R., De Freitas P.S., Scherf U. (2002). The Effect of Keto Defect Sites on the Emission Properties of Polyfluorene-Type Materials. Adv. Mater..

[22] Sahare P.D., Sharma V.K., Mohan D., Rupasov A.A. (2008). Energy transfer studies in binary dye solution mixtures: Acriflavine + Rhodamine 6G and Acriflavine + Rhodamine B. Spectrochim. Acta Part A Mol. Biomol. Spectrosc..

[23] Al-Asbahi B.A., Jumali M.H.H., Yap C.C., Flaifel M.H., Salleh M.M. (2013). Photophysical properties and energy transfer mechanism of PFO/Fluorol 7GA hybrid thin films. J. Lumin..

[24] Lakowicz J.R. (2013). Principles of Fluorescence Spectroscopy.

[25] Gilbert A., Baggott J.E. (1991). Essentials of Molecular Photochemistry.

[26] Schweitzer C., Schmidt R. (2003). Physical Mechanisms of Generation and Deactivation of Singlet Oxygen. Chem. Rev..

[27] Wu P., Brand L. (1994). Resonance Energy Transfer: Methods and Applications. Anal. Biochem..

[28] Mote U.S., Patil S.R., Bhosale S.H., Han S.H., Kolekar G.B. (2011). Fluorescence resonance energy transfer from tryptophan to folic acid in micellar media and deionised water. J. Photochem. Photobiol. B Biol..

